# Characterizing Early Adolescent Plate Waste Using the Mobile Food Record

**DOI:** 10.3390/nu9020093

**Published:** 2017-01-26

**Authors:** Chloe E. Panizza, Carol J. Boushey, Edward J. Delp, Deborah A. Kerr, Eunjung Lim, Krupa Gandhi, Jinan C. Banna

**Affiliations:** 1 University of Hawai’i at Manoa, Honolulu, HI 96822, USA; cpanizza@hawaii.edu; 2 University of Hawai’i Cancer Center, Honolulu, HI 96813, USA; cjboushey@cc.hawaii.edu; 3 Purdue University, West Lafayette, IN 47907-2035, USA; ace@ecn.purdue.edu; 4 Curtin University, Kent Street, Bentley, Perth, WA 6102, Australia; D.Kerr@curtin.edu.au; 5 University of Hawai’i John A. Burns School of Medicine, Honolulu, HI 96813, USA; lime@hawaii.edu (E.L.); kgandhi@hawaii.edu (K.G.)

**Keywords:** early adolescents, mobile food record, food waste, plate waste, eating behavior, portion size, dietary assessment, compost, recycle

## Abstract

This study aimed to assess the amount of plate waste and how plate waste was disposed by early adolescent girls using a mobile food record (mFR). Participants were girls nine to thirteen years residing in O’ahu, Hawai’i (*n* = 93). Foods selected and leftover were estimated using a three day mFR. Each leftover food was then classified as thrown into the trash, fed to a pet, eaten later, or other (e.g., composted). Repeated measures analyses of variance (ANOVA) were conducted and Tukey’s post-hoc test were used to adjust for multiple comparisons between times (breakfast, lunch, dinner, and snack) on leftover food and leftover food thrown into the trash. The percentage of food leftover and thrown into the trash was highest at lunch. The percentage of protein, grain, vegetables, fruit, and dairy leftover at lunch were unexpectedly low compared to previous studies. The median for percentage of food thrown into the trash at lunch was <5% for all food groups, and was consistently low across the day (<10%). Average energy intake was 436 kcal (±216) at lunch, and 80% of caregivers reported total household income as ≥$70,000. Studies in real-time using technology over full days may better quantify plate waste among adolescents.

## 1. Introduction

Plate waste—the edible portion of food that is selected and uneaten—has high associated financial and environmental costs [1,2], and contributes to the 133 billions pounds of food wasted by retailers and consumers in the United States every year [3]. In the school environment, adolescents in grades six to eight (age 12–14 years) were observed to discard 19% of entrees, 47% of fruit, 25% of milk, and 73% of vegetables [4]. The Environmental Protection Agency has reported that a large portion of edible waste ends up in landfills and contributes to approximately 16% of methane emissions in the United States [5]. 

Plate waste may also lead to an inadequate intake of nutrients. Children wasting a high percentage of the National School Lunch Program (NSLP) lunch are likely to replace these calories with higher calorie salty and sugary foods [4,6]. A study conducted in 3049 middle school students in Boston also found that while meals served to children met the United States Department of Agriculture’s (USDA) dietary requirements for the NSLP, children no longer met the requirements for iron, calcium, or vitamin C after accounting for plate waste [4]. In addition, these children then exceeded the recommended proportion of energy consumed from total fat and saturated fat [4].

While previous studies have indicated that plate waste in children and adolescents is substantial [7,8,9], several limitations exist with regard to prior efforts. First, research on plate waste is often limited to the examination of waste at lunch in the NSLP, and excludes an examination of waste in other settings [4,7,8,9]. Secondly, previous methods for assessing plate waste were resource- and time-intensive, and did not allow for data collection in real-time throughout a 24-h period. These include visual estimation of plate waste through observation [10,11,12], estimation through digital imaging [11,13,14], direct weighing of plate waste [4,11], and a combination of these methods [11]. New dietary assessment tools such as the image-based mobile food record (mFR) hold promise for evaluating plate waste in diverse settings [15,16]. The mFR allows 24-h food records to be completed in community dwelling environments through the use of an application on a mobile device, and has been shown to be feasible and acceptable in youth [15,16,17,18]. Participants take a “before image” of selected food and an “after image” of leftover food. A reference device—a color checkerboard known as a fiducial marker—is placed in each image for color and size estimation of food [16,17]. The mFR allows for automatic uploading of images to a cloud-based secure server when in 3G/4G/Wi-Fi range [18,19,20]. In addition, image-assisted dietary assessment methods—a combination of images and self-report—have proven to provide valid estimates of energy intake [19,20].

To determine appropriate solutions to the problem of plate waste and the associated nutritional implications, an accurate estimate of the magnitude of plate waste using technological tools that facilitate data collection over 24-h and in diverse settings is needed. During early adolescence, individuals become independent and increasingly consume foods away from the home [21], and plate waste in this group has been shown to be high [7,8,9]. Girls in this age group have been shown to waste significantly more energy, vegetables, chocolate milk, bread, protein, calcium, iron, and vitamin A than boys served a similar portion at lunch in the NSLP [7,8,9,22]. Further, in this stage of life, habits are developed that may persist into adulthood, making this an ideal time to intervene [23]. The objective of this study was to assess the amount of plate waste and how plate waste was disposed of in a multiethnic population of girls nine to thirteen years residing in O’ahu, Hawai’i using the mFR. 

## 2. Materials and Methods 

### 2.1. Materials Study Design

This cross-sectional study was conducted in O’ahu, Hawai’i. Data were collected between February and September 2015. The study was approved by the Institutional Review Board at the University of Hawai’i at Manoa.

### 2.2. Participants

Girls nine to thirteen years of age (*n* = 93) and their caregivers were recruited by posting flyers and giving presentations at local schools, youth centers, libraries, sporting clubs, and the University of Hawai’i, as well as through snowballing techniques. The study was advertised as a nutrition study investigating the eating behaviors of girls. The advertisement did not mention that plate waste behaviors would be investigated to reduce any study bias. The inclusion criteria were as follows: girl, aged nine to thirteen years, resident of O’ahu, Hawai’i, and caregiver willing to participate. Child assent and caregiver consent forms were completed prior to the start of data collection. 

### 2.3. Study Protocol

Participants attended two contact sessions of approximately 30–60 min in duration. The initial contact session was dedicated to training the participants to use the mFR to collect before and after images of all eating occasions over three days. Parents completed a demographic data form. The second session focused on reviewing the images, clarifying the content of the images, and obtaining anthropometric measures from girls. These sessions were held at the participant’s home, private rooms, on the university campuses, or at a local youth center. The participant and her caregiver were present at each session. The two researchers who conducted the sessions were both nutrition professionals. As an incentive for their involvement, each participant received $50 in gift cards to a state-wide supermarket chain.

### 2.4. Assessment of Food Waste 

Each participant was provided with an Apple iPod preloaded with the mFR app and two small square fiducial markers [18]. Participants were instructed to take a before image and an after image of everything they ate or drank excluding water using the mFR app over three days. A before eating image always had to be followed by an after eating image, as the mFR will not allow a participant to take a new before eating image unless an after eating image is recorded for the previous meal. Participants were trained to place one fiducial marker in the bottom left hand corner of every image. The marker was of known dimensions and color, and assisted the research dietitian to later estimate the volume of food and drinks in each image. Participants were also provided with a small booklet containing key information from the training presentation and a section for recording a written food record. If participants were unable to capture images of their food, they were encouraged to write these items in the notes application on the iPod or in the booklet. Upon completion of the training, participants chose three non-consecutive days to record using the mFR. To capture weekly variations, these included two weekdays and one weekend day over a one-week period. If iPods were not allowed at the participant’s school, caregivers were encouraged to ask for permission for the participant to take images of food and drinks consumed at school for the duration of the study. Participants were also instructed to take images of food and drink consumed at home and outside of home and school (e.g., at a friend’s house or a restaurant).

The second session (one week later) involved a review of images collected. In this session, the research dietitian reviewed all images on the mFR server, and notes on the participant’s iPod and in the booklet. These records, as well as a standard dietary recall script, model cups, plates, bowls, and measuring cups, and the fiducial marker in the images used as a reference device were used to estimate the quantity, brand, type, and ingredients of food selected and left over [15,18]. Probes were used to help participants recall any foods that were not recorded. Information on the time of eating was extracted from the image metadata (time and date stamps). The time stamp on the before eating image was documented as the time of consumption. Plate waste was recorded as the total amount of food left over. The mFR does not capture how leftovers are disposed; therefore, during this session, each leftover food item was further classified by the participant as thrown into the trash, fed to a pet, eaten later by the participant or someone else, or other (e.g., composted). All dietary information collected in the second session was entered into a plate waste recording template designed for this study. 

### 2.5. Anthropometry

Height and weight were collected during the second session using a calibrated scale and stadiometer using a standard protocol. Two measurements were recorded for each. Acceptable readings were those with a difference of equal to or less than 0.5 cm for height and 0.5 kg for weight. Additional readings were taken until these conditions were satisfied [24]. 

### 2.6. Data Analysis 

Analyses were limited to those participants with at least two days of recording. A record for the day required at least one or more recorded images or notes of an eating occasion. All images captured by the participant were included in their food record, including images taken without a fiducial marker. RapidCalc—a data entry program developed by the University of Hawai’i Cancer Center—was used for energy and nutrient analyses [25,26]. Three separate RapidCalc databases were created for food selected, food left over, and leftover food thrown into the trash. These three RapidCalc databases were then replicated and edited to provide data by time of day. Time of day was broken down into four periods: 6–9 a.m., 11–2 p.m., 5–8 p.m., and all other times. These represented breakfast, lunch, dinner, and snacks, respectively [27]. RapidCalc automatically calculated total energy (kcal), protein (g), grain (ounce), vegetable (cup), dairy (cup), and fruit (cup) per day for each dataset. 

Data on total energy from food selected, left over, and leftover food thrown into the trash for each time period and data on total protein, grain, vegetables, dairy, and fruit selected, left over, and leftover food thrown into the trash at lunch time were exported for further analysis. Percentage of food left over and percentage of food thrown into the trash were calculated for energy, protein, grain, vegetables, fruit, and dairy as follows:

- Percentage *y* left over = (total *y* left over/total *y* selected) *×* 100
- Percentage *y* thrown into the trash = (total *y* thrown into the trash/total *y* selected) *×* 100

Data on percentage energy left over and percentage energy thrown into the trash represented total food left over and total food thrown into the trash [28].

BMI *z*-score was calculated according to the Centers for Disease Control and Prevention BMI *z*-score guidelines for girls 5–19 years [29]. A body mass index (BMI) *z*-score of −3 or less represented severe thinness, −3 to −2 thinness, −2 to 1 healthy weight, 1 to 2 overweight, and greater than 2 obese [29]. Mother’s and father’s race was categorized according to the United States Census Bureau [30]. Participants’ race was recorded as their dominant race. Dominant race for each participant was calculated by averaging mother’s and father’s race and selecting the race of highest percentage. 

### 2.7. Statistical Methods 

Demographic variables were described as mean and standard deviation (SD) for continuous variables and frequencies and percentages for categorical variables. Percentage of food left over and percentage of food thrown into the trash were found to be skewed for each time period, and normality was violated by Shapiro–Wilk tests. Therefore, to reduce variance and approximate to normal distributions, log transformation was performed. Descriptive statistics were presented using the back-transformed value. Repeated measures analysis of variance (ANOVA) was conducted, and Tukey’s post-hoc test was used to adjust for multiple comparisons between times. Percentage of food left over and percentage of food thrown into the trash were highest at lunch; therefore, lunch time data were further split by food groups (protein, grain, vegetables, dairy, and fruit) and presented as descriptive data. A *p*-value < 0.05 was considered statistically significant, and SAS version 9.4 was used to conduct statistical analyses (SAS Institute, Cary, NC, USA).

## 3. Results

All 93 participants completed the study, and only seven participants had an mFR that did not meet the acceptable mFR criteria. Consequently, their data were removed from the final analysis, and the final sample size was 86 participants. The mean age of the group was 10.8 years (SD = 1.4), average energy intake was 1586 kcal (SD = 420) over the whole day and 426 kcal (SD = 216) at lunch, and 68 (82%) caregivers recorded a total household income of $70,000 USD or greater (Table 1).

Table 2 shows the percentage of energy left over and percentage of food thrown into the trash over the whole day and across time periods. The percentage of food left over was highest at lunch (Mean = 9.6%, SD = 10.5), and was significantly higher than the percentage of food left over at breakfast (Mean = 5.8%, SD = 8.8; *p =* 0.008) and snacks (Mean = 6.1%, SD = 8.7; *p* = 0.017), but not at dinner. The percentage of food thrown into the trash was also highest at lunch (Mean = 6.8%, SD = 9.2), and this was significantly higher than the percentage of food thrown into the trash at breakfast (Mean = 3.3%, SD = 6.2; *p* = 0.011), dinner (Mean = 3.3%, SD = 6.7; *p* = 0.005), and snacks (Mean = 2.3%, SD = 4.9; *p* = 0.0002). To investigate the robustness of this comparison, repeated measures ANOVAs were conducted using the original data with the three food records, time, and their interaction. The results on time were equivalent in magnitude and significance (not shown). 

Reviewing lunch time data by nutrient and food groups (Table 3), the percentages of protein, grain, and vegetables left over and percentages thrown into the trash were not normally distributed. For example, the median percentage of vegetable servings left over and thrown into the trash were 0 (interquartile range (IQR) = 0–17) and 0 (IQR = 0–14), respectively. The lower quartile and median for percentage left over and percentage thrown into the trash at lunch were <5% for all food groups. The upper quartile for percentage left over and percentage thrown into the trash at lunch were similar across protein, grain, and vegetables, at 16%–17% and 10%–14%, respectively. The upper quartile for percentage left over and percentage thrown into the trash for fruit and dairy was <5%.

In examining the relationship between percentage food left over and percentage food thrown into the trash, approximately 50% of the food left over was thrown into the trash at breakfast, dinner, and snacks, and 70% at lunch. At lunch, for the upper quartile, 60%–80% of leftover protein, grain, and vegetables were thrown into the trash.

## 4. Discussion

The percentages of food left over and percentages of food thrown into the trash were highest at lunch; however, less than 11% of food was left over across the day. There is no known acceptable percentage of plate waste; however, the United States has set a national target of reducing food waste from retailers and consumers by half by 2030, thereby reducing food waste from 31% to less than 15% [3]. Previous research on plate waste in the NSLP has also stated that plate waste of less than 12% is not unreasonable [7]. Plate waste was recorded across the day in this study and was not limited to the lunch period; therefore, results cannot be directly compared to these studies. However, plate waste still appears to be relatively low in this study. We also observed that approximately 50% of the leftover food was thrown into the trash at breakfast, dinner and snacks, and this increased to 70% at lunch. Despite plate waste being low in this study, the percentage of leftover food that was thrown into the trash was unacceptably high. Interventions aimed at reducing the amount of leftover food that is thrown into the trash should educate and encourage early adolescents to dispose of plate waste in alternative ways, such as composting if available or feeding leftovers to a pet, if safe to do so.

Splitting lunch time data by food groups revealed that the median quartiles of food left over and food thrown into the trash for protein, grain, vegetables, fruit, and dairy were low (<5%). The upper quartile of plate waste for fruit and dairy was close to zero and between 10% and 14% for protein, grain, and vegetables. Previous studies have found that fruit and vegetables were the most frequently wasted items, with plate waste up to 45% and 75%, respectively [7,28]. Again, these earlier studies were conducted on plate waste in the NSLP; however, plate waste of fruit and vegetables in this study was unexpectedly low. 

One factor that may account for why plate waste was lower in this group compared to previous plate waste studies conducted on adolescents in the NSLP is a difference in the amount of food selected. In the NSLP, schools are required to serve a lunch meal that has 550–650 kcal and 600–700 kcal for students in grades K-5 and grades 6–8, respectively [31]. In this study, participants selected a lunch with approximately 426 kcal. Consequently, the amount of food selected at lunch in this group appears to be lower than average. Selecting less food at lunch is only a concern if it results in a significant energy deficit and adolescents are no longer meeting their recommended daily energy intake. Only 8% of adolescents in the US perform the recommended amount of daily physical activity [32]. The recommended energy intake for girls 9–13 years of age who are sedentary is 1400–1600 kcal per day [33]. This group consumed on average 1406 kcal (SD = 392) over the whole day; therefore, selecting a lunch with fewer calories does not appear to be of concern, and may result in less plate waste. 

Socio-demographic characteristics may also account for the relatively lower amount of plate waste in this study. Previous studies reporting high fruit and vegetable plate waste in the NSLP were carried out in low-income schools, and participants received free or reduced priced lunch [4,7,8,9]. In comparison, 68 (80%) of the caregivers in this study reported that their total household income was $70,000 or more, rendering their children ineligible for free or reduced priced lunch [34]. Children with lower socio-economic status who receive a free school lunch may have more plate waste than those children with higher socio-economic status who do not receive a free school lunch. Previous NSLP studies have identified a number of factors that may contribute to waste of free or reduced-price lunch, including dislike of the taste, smell, or look of the food served, and the excessive amount of food served relative to age and gender [7]. Portion size, socio-demographic characteristics, the person preparing the food, and where food was served were not controlled for in this study; however, these should be recorded and accounted for in future plate waste studies. 

Historically, collecting plate waste data over 24 h was difficult, as available options for data collection involved being present to observe and/or weigh participants’ leftover food [4,7,35,36]. Previous research on plate waste in adolescents has been limited to a portion of the day (most commonly the lunch in the NSLP), and has excluded examination of plate waste during other times. In the current study, participants independently recorded plate waste using the mFR, and 86 (92%) participants completed an acceptable mFR. The mFR is a practical dietary assessment tool that allows participants to capture 24 h of plate waste data in a community dwelling setting without a researcher present, and may be used to further examine plate waste in a larger sample of youth. 

The current study has several limitations. First, some degree of estimation error occurred when calculating total energy intake. Food selected, left over, or thrown into the trash were not weighed, and quantities were estimated in the second interview using the mFR images, standard recall script, and measurement aids. Food items recorded in the plate waste template were matched as closely as possible to food items in RapidCalc; however, not all could be matched (e.g., unsweetened low fat soy milk was unavailable in RapidCalc, therefore was matched with low fat soy milk). Another limitation was that under- or over-reporting of food intake was not examined in this study. Recalling how leftover food was disposed in the second session may have also lead to under- or over-reporting of the amount of leftover food thrown into the trash. In addition, convenience sampling was used, and the study was cross-sectional; therefore, trends could not be examined over time. Further, the study only explored plate waste behaviors in female adolescents. Plate waste is known to be higher for females than males; however, the percentage of food left on the plate is still significant in males (~20%–45%) at lunch in the NSLP [37], and warrants further investigation. There was no control for portion size, socio-demographic factors, or who prepared and served the food (e.g., participants’ caregivers, the school, or a restaurant), and these are factors that may influence plate waste behaviors. Finally, although the analysis results showed the robustness of the method and results, we found a significant difference in percentage left over between the first and second day records (not shown). Further analysis is required to explore this observation.

## 5. Conclusions

Among a sample of adolescent girls, the percentage of food left over and the percentage of food thrown into the trash was highest at lunch; however, both were consistently low over the whole day (<10%). The percentage of protein, grain, vegetables, fruit, and dairy left over were also low over the whole day (<5%). Plate waste was unexpectedly low in this study when compared to previous plate waste studies conducted on the NSLP. This difference may be accounted for by smaller portion sizes and the higher income level of participants in the current study as compared to those in previous studies focused on low-income schools participating in the NSLP. Additionally, studies examining plate waste often focus exclusively on lunch on a single day. The low amount of plate waste found in the current study may be a result of data collection across days and eating occasions and capturing data outside of the NSLP. 

The current study was a cross-sectional examination of a convenience sample of 93 girls nine to thirteen years of age in O’ahu, Hawai’i, and results may not be generalized to plate waste behaviors in girls outside of this group. Larger studies are required to investigate 24-h plate waste in early adolescent girls and boys with the assistance of a mFR, controlling for portion size, sociodemographic characteristics, the person preparing the food, and where the food was consumed. Given that lunch time plate waste was lower than previously reported in studies investigating plate waste at lunch in the NSLP, further studies should examine factors contributing to plate waste in the NSLP and strategies to reduce this waste.

Almost all participants (92%) completed an acceptable mFR, documenting plate waste over a three-day period in a community dwelling environment. The mFR appears to be a practical tool to measure plate waste over the whole day with a low burden for both study participants and researchers. 

## Figures and Tables

**Table 1:** Characteristics of 86 girls nine to thirteen years living in O’ahu, Hawai’i who participated in the study and completed an acceptable mobile food record.

Variable	Total (*n* = 86)
Age (years), mean ± SD	10.8 ± 1.4
Height (m), mean ± SD	1.47 ± 0.11
Weight (kg), mean ± SD	41 ± 14
Body mass index (BMI) *z*-score, mean ± SD	0.05 ± 1.14
Total energy consumed (kcal) per day	1406 ± 392
Total energy plated (kcal) at lunch	426 ± 216
Race, *n* (%)	
White	29 (34%)
Asian	48 (56%)
Other ^a^	9 (10%)
Total household income, *n* (%)	
$0–$69,000	15 (18%)
$70,000 or more	68 (82%)
Mother’s education level, *n* (%)	
Graduated from a four-year college or university or less	35 (41%)
Attended and or completed graduate school or more	51 (59%)

^a^ Other race includes Native Hawaiian or Other Pacific Islander, American Indian, or Alaska Native, Black or African American, and Some Other Race [30].

**Table 2:** Percentage energy left over and percentage of food thrown into the trash over the day in 86 girls nine to thirteen years living in O’ahu, Hawai’i who participated in the study and completed an acceptable mobile food record.

	Mean (%) ± SD					
Energy	Whole Day	Breakfast	Lunch	Dinner	Snack	*p*-Value
Percentage left over ^a^	10.6 ± 9.6	5.8 ± 8.8	9.6 ± 10.5	7.4 ± 9.7	6.1 ±8.7	<0.02 ^c,d^
Percentage thrown into the trash ^b^	5.7 ± 6.4	3.3 ± 6.2	6.8 ± 9.2	3.3 ± 6.7	2.3 ± 4.9	<0.02 ^c,d,e^

^a^ Percentage y left over = (total y left over/total y selected) × 100; ^b^ Percentage y thrown into the trash = (total y thrown into the trash/total y selected) × 100. y represents either total energy (kcal), or protein (g), grain (ounce), vegetables (cup), fruit (cup), or dairy (cup); ^c^ Significant difference by Tukey’s post-hoc test: breakfast vs. lunch; ^d^ Significant difference by Tukey’s post-hoc test: lunch vs. snack; ^e^ Significant difference by Tukey’s post-hoc test: lunch vs. dinner. Repeated measures ANOVA was used on log-transformed outcome variables.

**Table 3:** Percentage of energy and food groups left over and percentage thrown into the trash at lunch 86 girls nine to thirteen years living in O’ahu, Hawai’i who participated in the study and completed an acceptable mobile food record.

Variable	Median Percentage Left Over ^a^ (IQR)	Median Percentage Thrown into the Trash ^b^ (IQR)
Energy	8 (0–16)	1 (0–13)
Protein	5 (0–17)	0 (0–12)
Grain	2 (0–17)	0 (0–10)
Vegetables	0 (0–17)	0 (0–14)
Fruit	0 (0–0)	0 (0–0)
Dairy	0 (0–4)	0 (0–0)

^a^ Percentage y left over = (total y left over/total y selected) × 100; ^b^ Percentage y thrown into the trash = (total y thrown into the trash/total y selected) × 100. y represents either total energy (kcal), or protein (g), grain (ounce), vegetables (cup), fruit (cup), or dairy (cup). IQR = Interquartile range.
